# Investigating the Phytochemical Composition, Antioxidant, and Anti-Inflammatory Potentials of *Cassinopsis ilicifolia* (Hochst.) Kuntze Extract against Some Oxidative Stress and Inflammation Molecular Markers

**DOI:** 10.3390/cimb46090573

**Published:** 2024-09-01

**Authors:** Emmanuel Mfotie Njoya, Lyndy J. McGaw, Tshepiso J. Makhafola

**Affiliations:** 1 Centre for Quality of Health and Living, Faculty of Health and Environmental Sciences, Central University of Technology, Bloemfontein 9300, Free State, South Africa; 2 Phytomedicine Programme, Department of Paraclinical Sciences, Faculty of Veterinary Science, University of Pretoria, Private Bag X04, Onderstepoort, Pretoria 0110, Gauteng, South Africa; lyndy.mcgaw@up.ac.za

**Keywords:** phytochemicals, radical scavenging potential, reactive oxygen species, cytokines, inflammation, oxidative stress

## Abstract

Oxidative stress is a key factor that activates several transcription factors and mediators involved in the inflammatory pathways responsible for the pathogenesis of many chronic diseases. Targeting the expression of these mediators represents a promising approach to preventing these diseases. *Cassinopsis ilicifolia* leaf infusion is traditionally used for treating conditions such as inflammation and pain relief. Thus, the present study assessed the antioxidant and anti-inflammatory activities of the hydroethanolic leaf extract of *C. ilicifolia* using in vitro and cell-based assays. As a result, *C. ilicifolia* extract exhibited the highest DPPH^•^ and ABTS^•+^ radical scavenging potential. At the same time, it weakly scavenged the Fe^3+^-TPTZ radical up to 200 µg/mL, thus suggesting a different antioxidant mechanism triggered during each assay. Additionally, *C. ilicifolia* extract inhibited NO production and 15-LOX activity with IC_50_ values of 21.10 µg/mL and 40.28 µg/mL, respectively. Further, *C. ilicifolia* extract was found to strongly inhibit ROS production in LPS-activated RAW 264.7 cells, and the study of its mechanism of action showed that it exerts its anti-inflammatory effect by downregulating the expression of inflammatory mediators such as IL-1β, TNF-α, and COX-2. Overall, *C. ilicifolia* extract showed consistent potency in all assays, and the analysis of its phytochemical profile led to the identification of 30 compounds, among which the most abundant were secologanic acid (1), chlorogenic acid (3CQA) (2), monotropein (3), chlorogenic acid (5CQA) (4), geniposidic acid (5), rutin (6), quercetin 3-galactoside (7), astragalin-7-rhamnoside (8), and minecoside (9) that are possibly responsible for its anti-inflammatory and antioxidant activities. Therefore, our findings suggested the potential use of *C. ilicifolia* as an alternative source for developing plant-based products against oxidative stress and inflammation-related conditions.

## 1. Introduction

Several non-communicable diseases, including type II diabetes, arthritis, cancer, and cardiovascular and neurodegenerative diseases, have been associated with oxidative stress and inflammation [1]. Since one can cause the other and vice-versa, oxidative stress and inflammation are interconnected [2]. Oxidative stress is caused by a disproportion between the body’s antioxidant systems’ capacity to eliminate free radicals and their excessive production. Long-term persistence of this condition may cause inflammation because of the immune system’s defensive reaction to the imbalanced control of free radicals [2,3]. More specifically, innate cells, including macrophages, are recruited to inflammatory sites, and trigger the release of inflammatory mediators such as prostaglandin E_2_ (PGE_2_), nitric oxide (NO), cyclooxygenase-2 (COX-2), and pro-inflammatory cytokines (IL-1β, IL-6, TNF-α, etc.) [4,5]. Furthermore, the overproduction of these pro-inflammatory mediators enhances the inflammatory response, therefore accelerating the development of pathogenic inflammation and chronic diseases via the activation of the transcription factor, nuclear factor-kappa B (NF-κB) [6]. On the other hand, immunocompetent cells, such as macrophages, activate the lipoxygenase (LOX) pathway, and numerous studies have documented the significance of 12/15-LOX in oxidative and inflammatory responses [7,8]. Thus, using plant-based products to target free radicals or inflammatory mediators and inhibiting 12/15-LOX is a promising strategy for the development of novel medicines against the above-mentioned chronic disorders.

The use of medicinal plants to prevent or treat a wide range of diseases is common in South Africa. The public and medical professionals are accepting the benefit of medicinal plants and their derivative products due to numerous research studies that have been conducted to examine their therapeutic efficacy and mechanisms that underlie their positive effects on health and quality of life. The therapeutic potential of medicinal plants is mostly attributed to the presence of bioactive secondary metabolites, which may act individually, additively, or synergistically to improve human health [9,10]. Furthermore, research indicates that 35–50% of approved medications over the past 30 years have either directly or indirectly derived from natural products [11,12]. This suggests that medicinal plants can be important sources of novel bioactive compounds or precursors for the synthesis of new drugs. Medicinal plants are generally thought to be effective at scavenging free radicals and possessing a variety of therapeutic properties, including anti-inflammatory, anticancer, and antioxidant activities [13]. The free radical scavenging property is thought to be related to the presence of phenolics and flavonoids, which can inhibit the production of inflammatory mediators or regulate the expression of various signaling pathways. Therefore, plant-based products seem to be an important source of free radical scavengers that protect against oxidative stress and inflammation. *Cassinopis ilicifolia* is a South African indigenous plant from the family of Icacinaceae, and its leaf and bark infusions are traditionally used for treating stomachache problems, tuberculosis, inflammation of the ear, and pain relief [14,15]. To date, *C. ilicifolia* ethanolic extract has been found to have antimicrobial activity against *Staphylococcus aureus* with an MIC value of 0.39 mg/mL and anthelmintic activity against *Caenorhabditis elegans* with an MLC value of 0.033 mg/mL. Additionally, the anti-inflammatory activity of *C. ilicifolia* ethanolic extract was assessed against cyclooxygenases (COX), with higher inhibitory activity against COX-2 compared to that of COX-1 [15]. These results suggested the inhibitory potential of this extract against other pro-inflammatory mediators and oxidative stress markers, known as common causes of several chronic diseases. Moreover, the phytochemical composition of *C. ilicifolia* is not fully known. Therefore, the present study aims to investigate the antioxidant and anti-inflammatory potentials of *C. ilicifolia* and determine the phytochemicals that might justify its pharmacological properties. In this regard, we used in vitro methods and cell-based assays on LPS-activated RAW 264.7 macrophages to determine its radical scavenging capacity, its preventive effect on the generation of reactive oxygen species (ROS), and its modulatory effect on the expression of inflammatory mediators. The outcomes of this investigation may shed light on the beneficial effects of *C. ilicifolia* on humans and open further directives toward the development of natural products against oxidative stress and inflammation-related conditions.

## 2. Materials and Methods

### 2.1. Plant Material and Method of Extraction

Fresh leaves of *C. ilicifolia* were collected at the Walter Sisulu National Botanical Garden, Gauteng (South Africa). A herbarium specimen was prepared, and identification was made by Ms. Magda Nel and Mrs. Elsa van Wyk of the HGWJ Schweickerdt Herbarium (PRU), University of Pretoria, with the identification number PRU 119039. Each plant’s leaves were dried in a well-ventilated room at ambient temperature, and a fine powder was obtained after grinding the dried leaves. After that, 50 g of leaf powder was macerated in 500 mL of ethanol/water (80:20) with constant stirring for 48 h at room temperature. Whatman No. 1 filter paper was used to filter, and the filtrate was collected into beakers previously weighed. After drying the filtrate under a cold air stream, residue named crude extract was obtained, and the beaker was weighed again to deduce the quantity extracted, and the yield of extraction was computed as a percentage. Until they were used, the dried extract was kept in a 4 °C cold chamber.

### 2.2. Antioxidant Assays

#### 2.2.1. Azino-bis (3-Ethylbenzothiazoline-6-Sulfonic Acid) (ABTS^•+^) Radical Scavenging Assay

The technique used to determine the potential of crude extracts to scavenge ABTS^•+^ radical was outlined by Miller et al. [16] and adjusted by Re et al. [17]. An ABTS solution (7 mM in methanol) and a potassium persulfate solution (2.45 mM in methanol) were mixed at ambient temperature for at least 16 h to form ABTS^•+^ radical cation. The absorbance of the generated radical ABTS^•+^ was assessed at 734 nm on a multi-mode microplate reader, SpectraMax iD3 (Molecular Devices, San Jose, CA, USA), and calibrated to 0.70 ± 0.02. Next, on a 96-well microtiter plate, 1 mg/mL of *C. ilicifolia* extract (40 µL) was serially diluted with methanol, and then ABTS^•+^ radical cation (160 µL) was added. After 5 min of dark incubation at ambient temperature (25 °C), the absorbance readings were recorded against a blank (extract containing methanol without ABTS^•+^) at 734 nm using a multi-mode microplate reader, SpectraMax iD3 (Molecular Devices, San Jose, CA, USA). Methanol plus radical cation ABTS^•+^ was used as the negative control, while ascorbic acid (0–200 µg/mL) was utilized as the positive control. Formula (1) below was used to calculate the percentage of ABTS^•+^ radical scavenging potential at each dosage. The inhibitory concentrations (IC_50_) values were then obtained by plotting a non-linear regression curve of percentage ABTS^•+^ radical scavenging potential against the logarithm of various extract dosages.

Radical scavenging capacity (%) = [(A_0_ − A_1_)/A_0_] × 100 (1)

A_0_ is the absorbance of the negative control and A_1_ is the absorbance of extract plus the radical cation ABTS^•+^.

#### 2.2.2. Diphenyl-1-Picrylhydrazyl (DPPH^•^) Radical Scavenging Assay

The technique outlined by Brand-Williams et al. [18] was used to assess the extracts’ capacity to scavenge DPPH^•^ radical. In summary, on 96-well microtiter plates, 1 mg/mL of *C. ilicifolia* extract (40 µL) was serially diluted with methanol, and a DPPH^•^ solution (160 µL, 25 µg/mL in methanol) was added. Using a multi-mode microplate reader SpectraMax iD3 (Molecular Devices, San Jose, CA, USA), the absorbance readings were recorded at 517 nm against a blank (extract containing methanol without DPPH^•^) after dark incubation of plates at ambient temperature (25 °C) for 30 min. The negative control was made of methanol plus DPPH^•^, while the positive control was constituted of a serial dilution of ascorbic acid (0–200 µg/mL). Using the preceding formula (1), the DPPH^•^ scavenging potential was computed at each dosage, and the IC_50_ values were determined as previously indicated.

#### 2.2.3. Ferric Reducing Antioxidant Power (FRAP) Assay

The method outlined by Benzie and Strain [19] was utilized to assess the antioxidant potential in a colorimetric redox reaction in which iron-tripyridyltriazine (Fe^3+^-TPTZ) is reduced at low pH to a dark blue iron-tripyridyltriazine complex (Fe^2+^-TPTZ) [20]. Briefly, 10 mg/mL of *C. ilicifolia* extract (50 µL) was serially diluted to obtain different concentrations (1.56–200 µg/mL), or 50 µL of 200 µg/mL gallic acid (Merck, Darmstadt, Germany) were separately incubated for 30 min at 50 °C with 50 µL of potassium ferricyanide (1%; prepared in 0.2 M sodium phosphate buffer, pH 6.6). Ascorbic acid (0–200 µg/mL) used as the positive control was tested in similar conditions as crude extract. Subsequently, 50 µL of trichloroacetic acid (10%), 40 µL of distilled water, and 10 µL of ferric chloride (0.1%) were added, respectively. After that, the absorbance readings were recorded at 700 nm against a blank (extract containing sodium phosphate buffer) using a multi-mode microplate reader, SpectraMax iD3 (Molecular Devices, San Jose, CA, USA). The FRAP activity was then reported as the inhibitory percentage of gallic acid (200 µg/mL).

### 2.3. Anti-Inflammatory Assays

#### 2.3.1. Soybean 15-Lipoxygenase Inhibitory Assay

The test was conducted according to the protocol established by Pinto et al. [21], with a few amendments adapted to the 96-well microtiter plate format. The principle of this test is based on the formation of a Fe^3+^/xylenol orange complex whose absorption maximum is observed at 560 nm. On a 96-well microtiter plate, 10 mg/mL of *C. ilicifolia* extract (20 µL) was serially diluted with distilled water, and 40 µL of soybean 15-lipoxygenase (15-LOX) (200 UI/mL) (Merck, Darmstadt, Germany) was added. The positive control, gallic acid (0.78 to 100 µg/mL), was used as the standard inhibitor of 15-LOX, while the negative control was made of DMSO at 5% (*v*/*v*). The microtiter plates were then incubated at ambient temperature (25 °C) for 5 min. Subsequently, 140 μM of linoleic acid (40 µL) used as substrate prepared in Tris-HCl buffer (50 mM, pH 7.4) was added, followed by dark incubation of plates at ambient temperature (25 °C) for 20 min. The assay was completed by adding 100 µL of FOX reagent used as the stop solution, which is made of 30 mM sulfuric acid, 100 μM xylenol orange, and 100 μM ferrous II sulfate, prepared in methanol/water (9:1). The absorbance readings were measured at 560 nm on a multi-mode microplate reader, SpectraMax iD3 (Molecular Devices, San Jose, CA, USA), after dark incubation of microtiter plates at ambient temperature (25 °C) for 30 min. The blanks were prepared in the same manner as assayed samples, with the exception that FOX reagent was added after the substrate. Formula (2) below was used to calculate the 15-LOX inhibitory activity, and the software GraphPad Prism 6.0 (GraphPad Software, Inc., San Diego, CA, USA) was used to plot a non-linear regression curve of 15-LOX inhibitory activity against the logarithm (log10) of various dosages. This allowed for the determination of IC_50_ values for both extracts and gallic acid.

15-LOX inhibitory activity (%) = 100 − [(A_0_ − A_1_) − (A_2_ − A_1_)] × 100 (2)

A_0_ is the absorbance of assayed samples, A_1_ is the absorbance of the blank, and A_2_ is the absorbance of the negative control (DMSO 5%).

#### 2.3.2. Culture of RAW 264.7 Murine Macrophages and Cytotoxicity Assay

RAW 264.7 macrophages were purchased from the American Type Culture Collection (ATCC^®^ TIB-71^™^) (Rockville, MD, USA), and Roswell Park Memorial Institute (RPMI) 1640 medium (Cytiva Hyclone, Logan, UT, USA) supplemented with 10% FBS and 1% penicillin/streptomycin (P/S) was used for the maintenance of cells in a CO_2_ incubator (Nϋve, Ankara, Turkey) at 37 °C in a humidified environment containing 5% CO_2_ and 95% air. The cells were trypsinized at 70–80% confluency using trypsin/EDTA (0.25%; Cytiva Hyclone, Logan, UT, USA) and split at a ratio of 1:5 for the next sub-culturing. Using an automated cell counter (NanoEntek, Seoul, South Korea), the percentage of cell viability was assessed with 0.4% trypan blue (Cytiva Hyclone, Logan, UT, USA). For our investigations, only cell suspensions with a percentage of cell viability greater than 90% were used.

The cytotoxic effect of the *C. ilicifolia* extract was assessed using the 3-(4,5-dimethythiazol-2-yl)-2,5-diphenyl tetrazolium bromide (MTT) assay [22]. On a 96-well microtiter plate, RAW 264.7 cells (20,000) were seeded per well, and the cells were allowed to adhere overnight at 37 °C in a humidified environment containing 5% CO_2_ and 95% air. After, the cells were exposed to *C. ilicifolia* extract (3.125–100 µg/mL) for 24 h and further incubated at 37 °C in a humidified environment containing 5% CO_2_ and 95% air. Next, the culture medium including tested extract was removed with an aspirator and replaced with 200 µL of newly prepared medium and 30 µL of a 5 mg/mL thiazolyl blue tetrazolium bromide (Melford, Ipswich, UK) prepared in phosphate-buffered saline. The microtiter plates were further incubated at 37 °C in a humidified environment containing 5% CO_2_ and 95% air for 4 h, after which the culture medium was carefully aspirated from the wells. After dissolving the formazan crystals in 50 µL of DMSO at room temperature for 15 min in the dark, the absorbance readings were recorded at 570 nm using a multi-mode microplate reader, SpectraMax iD3 (Molecular Devices, San Jose, CA, USA).

#### 2.3.3. Nitric Oxide Production Inhibitory Assay

Nitric oxide (NO) generated by murine RAW 264.7 murine macrophages was assessed using the Griess reagent (Sigma Aldrich, Hamburg, Germany) after 24 h of exposure with 1 µg/mL of lipopolysaccharide (LPS) in the presence of *C. ilicifolia* extract or quercetin used as a positive control [23]. DMSO (0.5%) and LPS treated cells were used as the negative control. After exposure, cell supernatant (100 µL) from each well of the 96-well microtiter plates was passed on a new 96-well microtiter plate, and Griess reagent (100 µL) was added. The plates were left at room temperature (25 °C) in the dark for 15 min, and the absorbance readings were recorded at 540 nm on a multi-mode microplate reader SpectraMax iD3 (Molecular Devices, San Jose, CA, USA). The amount of nitrite was determined from a calibration curve (y = 0.0029x + 0.0114, R^2^ = 0.994) obtained with sodium nitrite (0–100 µM) used as standard. Based on each extract’s capacity to suppress NO release by LPS-activated RAW 264.7 macrophages relative to the negative control (DMSO (0.5%) and LPS-treated cells without extract), the percentage of NO production inhibitory activity was determined at each tested concentration. 

### 2.4. Quantification of the Levels of Inflammatory Mediators in LPS-Activated RAW264.7 Cells

The enzyme-linked immune sorbent assay (ELISA) method was used according to the manufacturers’ guidelines for commercial kits. Murine RAW 264.7 cells (500,000) were seeded per well into 6-well microtiter plates, and they were left to adhere overnight at 37 °C in a humidified environment containing 5% CO_2_ and 95% air. Before the addition of LPS (200 ng/mL) for 24 h, the cells were pre-treated with *C. ilicifolia* extract (12.5, 25, and 50 µg/mL), quercetin at 25 µM (positive control), and DMSO at 0.5% (negative control) for 2 h at 37 °C in a humidified environment containing 5% CO_2_ and 95% air. Following that, cells were rinsed twice with PBS (Cytiva Hyclone, Logan, UT, USA) and detached using trypsin-EDTA (0.25%; Cytiva Hyclone, Logan, UT, USA). By replicating thrice, the freeze–thaw cycles in liquid nitrogen (−194 °C) and a water bath (37 °C), the cell lysates were collected in PBS with a phosphatase inhibitor and EDTA-free Pierce protease tablets (ThermoFisher Scientific, Lenexa, KS, USA). The cell homogenates were centrifuged at 13,000× *g* at 4 °C, and the supernatants were aspirated and used for the estimation of the levels of inflammatory mediators such as interleukins (IL-1β, IL-10), tumor necrosis factor (TNF-α), and cyclooxygenase-2 (COX-2) using respectively Elabscience^®^ Mouse IL-1β, IL-10, TNF-α, and COX-2 ELISA kits (Elabscience Biotechnology Inc., Houston, TX, USA) in accordance per the manufacturer’s guidelines.

### 2.5. Determination of Intracellular Reactive Oxygen Species Levels

On black/clear bottom microtiter plates, RAW 264.7 murine macrophages (10,000) were seeded and left to adhere overnight at 37 °C in a humidified environment containing 5% CO_2_ and 95% air. These cells were then pre-treated with *C. ilicifolia* extract at non-toxic concentrations for 2 h, followed by exposure or not to 200 ng/mL LPS for 24 h at 37 °C in a humidified environment containing 5% CO_2_ and 95% air. Subsequently, the intracellular ROS levels were assessed using the fluorescent probe 2′,7′dichlorodihydrofluorescein diacetate (DCFH-DA) (Sigma-Aldrich, Hamburg, Germany) [24]. Briefly, the cells were washed once with PBS, and a free-FBS fresh culture medium containing 10 µM of DCFH-DA was added, followed by incubation for 30 min under standard culture conditions. The fluorescent probe was aspirated and replaced with fresh PBS, and the fluorescence of cells was determined at 485 nm (excitation) and 535 nm (emission) using a multi-mode microplate reader, SpectraMax iD3 (Molecular Devices, San Jose, CA, USA). The intracellular ROS levels were computed as the percentage of negative control cells. Using an excitation/emission filter (480/535 nm), images were taken on a 40X objective with a fluorescence microscope (Leica Microsystems GmbH, Wetzlar, Germany) coupled to a Flexacam C1 camera (Leica Microsystems GmbH, Wetzlar, Germany). 

### 2.6. Phytochemical Analysis and Tentative Identification of Compounds in Extract

#### 2.6.1. Total Phenolic Content

The Folin–Ciocalteu colorimetric method described by Singleton et al. [25] and adapted to a 96-well microtiter plate by Zhang et al. [26] was utilized to assess the total phenolic content (TPC) of *C. ilicifolia* crude extract. Briefly, the reaction solution was obtained by sequentially mixing 5 mg/mL of *C. ilicifolia* extract (20 µL), Folin–Ciocalteu reagent (100 µL; Folin–Ciocalteu/distilled water (1:9)), and 7.5% (*w*/*v*) Na_2_CO_3_ solution (80 µL). It was then left in the dark for 30 min at ambient temperature (25 °C). Using a multi-mode microplate reader, SpectraMax iD3 (Molecular Devices, San Jose, CA, USA), the absorbance readings of the mixture were recorded at 765 nm in comparison to ethanol used as a blank. A gallic acid calibration curve (0–100 mg/L; y = 0.208x + 0.129; R^2^ = 0.998) was used to transform the absorbance readings into milligrams of gallic acid equivalent (GAE) per gram of dry extract.

#### 2.6.2. Total Flavonoid Content

The aluminum chloride colorimetric method was used to quantify the total flavonoid content (TFC) of crude extracts [27]. Using test tubes, the reaction mixture was composed by combining 0.3 mg/mL of *C. ilicifolia* extract (2 mL), 10% aqueous aluminum chloride hexahydrate solution (0.1 mL), 1M potassium acetate (0.1 mL), and deionized water (2.8 mL). After homogenization, this mixture was left for 10 min at ambient temperature (25 °C), and 200 µL of each mixture was passed on a 96-well microtiter microplate to read the absorbance at 415 nm using a multi-mode microplate reader, SpectraMax iD3 (Molecular Devices, San Jose, CA, USA). Using a quercetin calibration curve (0–0.1 mg/mL; y = 7.588x + 0.241; R^2^ = 0.999), the absorbance readings were converted into milligrams of quercetin equivalents (QE) per g of dry extract.

#### 2.6.3. Liquid Chromatography–Mass Spectrometric (LC–MS) Analysis

Forty milligrams of *C. ilicifolia* extract was dissolved in 2 mL of methanol, spun down, and then 10-fold diluted with methanol before passing to glass vials for high-resolution LC–MS analysis, which was carried out using a Waters Cyclic Quadrupole time-of-flight (qTOF) mass spectrometer (MS) coupled to a Waters Acquity ultra-performance liquid chromatograph (UPLC) (Waters, Milford, MA, USA). The eluate from the column first passed through a photodiode array (PDA) detector before reaching the mass spectrometer, allowing simultaneous detection of ultraviolet (UV) and MS spectra. Negative mode electrospray ionization was used with a desolvation temperature set at 275 °C, a cone voltage of 15 V, and desolvation gas at 650 L/h, and the remaining MS conditions were optimized for high resolution and sensitivity. Data were collected by scanning from *m*/*z* 150 to 1500 *m*/*z* in both MSE and resolution modes. MS data from two channels were acquired in MSE mode: one at a low collision energy (4 V) and the second using a collision energy ramp (40−100 V) to generate fragmentation data. Leucine enkaphalin was used as reference mass for accurate determination of mass, and the instrument was calibrated with sodium formate. Two microliters of crude extract were injected, and separation was carried out on a Walters HSS T3, 2.1 × 100 mm, 1.7 μm column. The flow rate was 0.3 mL/min, and the column temperature was kept at 55 °C. The mobile phase consisted of water containing 0.1% formic acid (solvent A) and acetonitrile containing 0.1% formic acid (solvent B). The gradient started at 100% solvent A for 1 min and changed linearly to 28% solvent B over 22 min. It then moved to 40% solvent B over 50 s, followed by a wash step of 1.5 min at 100% solvent B, and ended by re-equilibration to initial conditions for 4 min. A calibration curve for chlorogenic acid (3CQA) was established by injecting 3CQA at different concentrations (0.5 to 100 mg/L) obtained under similar instrumental conditions as the injected extract. 

MS-DIAL and MS-FINDER (RIKEN Center for Sustainable Resource Science: Metabolome Informatics Research Team, Kanagawa, Japan) were used for data analysis after compression, centroiding, and application of reference mass correction [28,29]. In fact, functions 1 (unfragmented channel) and 2 (fragmented channel) of the Waters MS data were processed by MS-DIAL to produce MS1 and MS2 spectra with extraction of peak height intensity data. Since calibration standards are not available for all compounds, a semi-quantitative method was used to transform the peak height intensity to relative concentration by interpolating on a calibration curve for chlorogenic acid (3CQA). Each deconvoluted feature (alignment in MS-DIAL), together with its associated MS1 and MS2 spectra, was exported from MS-DIAL to MS-FINDER for a tentative identification of compounds. Based on the accurate mass elemental compositions, possible compounds were identified from available databases and then subjected to in silico fragmentation. By comparing the in silico and measured spectra, a score (out of 10) was attributed to each potential compound match, with the highest score showing the most possible identification being a minimum score of 4.

### 2.7. Statistical Analysis

Three replicate experiments were conducted for each assay, and the data are presented as the mean ± SEM (standard error of mean) values. The software GraphPad Prism 6.0 (GraphPad Software Inc., San Diego, CA, USA) was used for statistical analysis. One-way analysis of variance (ANOVA) and Student–Newman–Keuls or Dunnett’s tests were used to compare the data between tested samples and/or positive controls. A *p*-value less than 0.05 indicated a significant difference in the results.

## 3. Results and Discussion

### 3.1. Antioxidant Activity of C. ilicifolia Crude Extract

The antioxidant activity of *C. ilicifolia* hydroethanolic leaf extract was examined using ABTS^•+^, DPPH^•^, and Fe^3+^-TPTZ radical scavenging assay techniques. The combination of these assays is very important as they can be used to test lipophilic, hydrophilic, and hydrophobic antioxidants. The ABTS^•+^ scavenging assay can be used to measure both lipophilic and hydrophilic antioxidants, while the FRAP assay only assesses hydrophilic antioxidants, and the DPPH^•^ scavenging assay only applies to hydrophobic compounds [30,31]. As presented in Table 1, *C. ilicifolia* extract had good antioxidant potentials in the DPPH and ABTS assays, with IC_50_ values of 31.61 µg/mL and 21.29 µg/mL, respectively (Figure S1). It is known that a lower IC_50_ value suggests a higher antioxidant power. As such, *C. ilicifolia* extract was not as effective as ascorbic acid (positive control) that showed strong radical scavenging capacities in all assays. Further, the scavenging potency of extracts changed according to the radical involved, and this is illustrated by the fact that the IC_50_ values in the ABTS assay were lower than those in the DPPH assay, thus suggesting different polarity of antioxidant compounds in each assay. Likewise, extracts active in both the DPPH and ABTS assays weakly scavenged the Fe^3+^-TPTZ radical, and the IC_50_ values were extrapolated to be higher than 200 µg/mL. These data imply either a different antioxidant mechanism compared to the ABTS^•+^ and DPPH^•^ assays or a different polarity of antioxidant compounds in the FRAP assay. This can be interpreted by the fact that an antioxidant compound within an extract may have different scavenging mechanisms of action on different radicals, especially considering that antioxidant assays depend on the polarity of active compounds and reaction mechanism [32].

### 3.2. Anti-Inflammatory Activity of C. ilicifolia Crude Extract

#### 3.2.1. Lipoxygenase Inhibitory Activity

The anti-inflammatory activity of *C. ilicifolia* crude extract was first evaluated in vitro against 15-LOX enzymatic activity. This enzyme is known to regulate inflammatory responses through the release of pro-inflammatory lipid mediators such as leukotrienes, and several reports have proven its implication in the development of various chronic diseases [7]. Inhibition of 15-LOX suggests a reduction of the release of lipid mediators, and this will lessen the deleterious effects of inflammation. Therefore, the 15-LOX inhibitory activity of *C. ilicifolia* crude extract was assessed using the xylenol orange iron oxidation assay. Data obtained showed that *C. ilicifolia* extract inhibited the 15-LOX enzymatic activity in a dose-dependent manner (Figure S2) with an IC_50_ value of 40.28 µg/mL. When compared to gallic acid, which was used as a positive control and is known as a potent 15-LOX inhibitor (IC_50_ value of 22.08 ± 1.96 µg/mL), *C. ilicifolia* extract was less efficient than gallic acid, a pure compound, and this may be justified by the low concentrations of bioactive compounds within *C. ilicifolia* crude extract. However, the *C. ilicifolia* extract can be assumed to be a potential source of compounds with 15-LOX inhibitory potential. The isolation and characterization of such compounds responsible for the efficacy of this extract against 15-LOX enzymatic activity is therefore important, as this may result in the discovery of new drug candidates against inflammatory-related diseases.

#### 3.2.2. Cytotoxic Potency of *C. ilicifolia* Extract on RAW 264.7 Murine Macrophages

**Figure 1:**
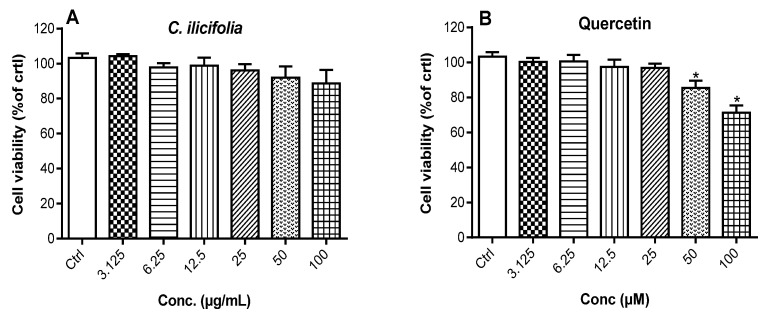
Cytotoxicity potency of *C. ilicifolia* hydroethanolic extract (**A**) and quercetin (**B**) on RAW 264.7 macrophages. These cells were exposed to increasing concentrations *C. ilicifolia* and quercetin for 24 h and the percentage of cell viability was assessed using MTT assay. Data are represented as percentage of 0.5% DMSO-treated cells (Ctrl). Each bar depicts the mean ± SEM of three replicates (*n* = 3). One-way ANOVA combined with Dunnett’s test was used for data analysis. * *p* < 0.05 vs. Ctrl.

The cytotoxic potency of *C. ilicifolia* extract was determined on RAW 264.7 murine macrophages by exposing these cells to increasing concentrations of extract and quercetin for 24 h, followed by an evaluation of cell viability using the MTT assay, a method that measures the capacity of live cells to transform MTT absorbed by cells into formazan crystals using dehydrogenase and oxidoreductase enzymes [22,33]. The negative control comprised 0.5% DMSO-treated cells, which was considered 100% cell viability, and the cytotoxic potency of tested samples was expressed as percentage of 0.5% DMSO treated cells (negative control). *C. ilicifolia* extract slightly reduced the cell viability at 100 µg/mL, but this cytotoxic potency was not significantly distinct from that of the negative control (Ctrl) (Figure 1). Quercetin was found to be significantly (*p* < 0.05) cytotoxic at higher concentrations tested (50 and 100 µM). Thus, non-cytotoxic doses of tested samples were used in our future experiments on RAW 264.7 murine macrophages.

#### 3.2.3. Nitric Oxide Production Inhibitory Effects of *C. ilicifolia* Crude Extract

**Figure 2:**
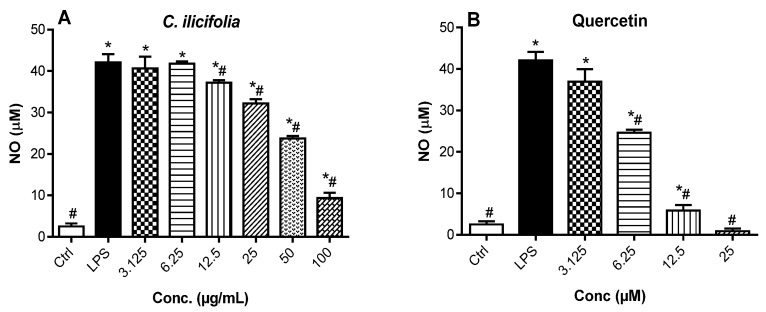
Nitric oxide (NO) released by RAW 264.7 macrophages after pre-treatment with *C. ilicifolia* hydroethanolic extract (**A**) and quercetin (**B**). RAW 264.7 cells were exposed to non-toxic concentrations of *C. ilicifolia*, and quercetin for 2 h, followed by 24 h of LPS (1 µg/mL) activation. The release of NO was assessed by quantifying the levels of nitrite in cell supernatants using the Griess reagent. Each bar depicts the mean ± SEM of three replicates (*n* = 3). One-way ANOVA combined Dunnett or Student–Newman–Keuls’s tests were used for data analysis. * *p* < 0.05 vs. Ctrl. ^#^ *p* < 0.05 vs. LPS.

Nitric oxide (NO) is a pro-inflammatory mediator synthesized by the inducible nitric oxide synthase (iNOS), involved in the regulation of inflammatory responses, and its excessive production can instigate oxidative stress [34,35]. As a result, iNOS functions to generate the free radical NO as a product of its enzymatic activity. Therefore, measuring the NO production in cells automatically correlates with the quantification of iNOS expression. The potential anti-inflammatory activity of *C. ilicifolia* extract was investigated by assessing the NO production inhibitory effect on LPS-activated RAW 264.7 murine macrophages. The measurement of NO production in cell supernatants was carried out using Griess reagent. It was found that LPS treatment of RAW 264.7 murine macrophages significantly (*p* < 0.05) boosted NO release compared to non-treated cells (Ctrl), and *C. ilicifolia* extract and quercetin prevented LPS-generated NO release in a dose-dependent manner (Figure 2). Quercetin, a known anti-inflammatory compound, inhibited NO release with an IC_50_ value of 7.08 µM, while *C. ilicifolia* extract had an IC_50_ value of 21.10 µg/mL (Figure S3). As *C. ilicifolia* extract showed significant anti-inflammatory effects by strongly preventing NO release in LPS-activated RAW 264.7 macrophages, the measurement of iNOS expression after exposure to *C. ilicifolia* extract will be tested in future experiments as both molecular markers are important contributors to cellular oxidative stress and inflammation. However, the modulatory effect of *C. ilicifolia* extract was tested on the expression of cytokines in subsequent experiments.

### 3.3. Regulatory Effect of C. ilicifolia Extract on the Levels of Inflammatory Mediators

**Figure 3:**
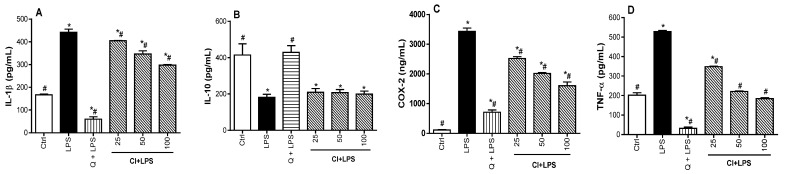
Regulatory impact of *C. ilicifolia* and quercetin on the expression of IL-1β (**A**), IL-10 (**B**), COX-2 (**C**), and TNF-α (**D**). RAW 264.7 cells were exposed to non-toxic concentrations of *C. ilicifolia* (CI), and quercetin (Q) at 25 µM for 2 h, succeeded by 24 h treatment with LPS (200 ng/mL). Each bar represents the mean ± SEM of two replicates (*n* = 2). One-way ANOVA combined Dunnett or Student–Newman–Keuls’s tests were used for data analysis. * *p* < 0.05 vs. Ctrl. ^#^ *p* < 0.05 vs. LPS.

To confirm the anti-inflammatory potential of *C. ilicifolia* extract, we investigated its potential to modulate the levels of pro-inflammatory mediators (COX-2, IL-1β, and TNF-α) and IL-10 anti-inflammatory cytokines on LPS-activated RAW 264.7 murine macrophages. Pro-inflammatory cytokines, namely TNF-α and IL-1β, are implicated in the inflammatory responses through the activation of immune cells and increasing the release of prostaglandins, while IL-10 is produced to terminate or alleviate an uncontrolled inflammatory response either by directly suppressing the effect of pro-inflammatory cytokines or by intercepting the synthesis of cytokines [36,37]. In addition, COX-2 is an enzyme stimulated in response to inflammatory stimuli, which play a crucial role in converting fatty acids to prostaglandins, which support the development of inflammation [38]. As such, the regulation of the levels of these inflammatory mediators is an effective line of action to control the inflammatory process. In this regard, the levels of inflammatory mediators were measured in cell lysates obtained from cells pretreated with non-toxic concentrations of bioactive extracts for 2 h, followed by exposure to LPS for 24 h. It was observed that the LPS exposure of RAW 264.7 macrophages significantly (*p* < 0.05) upregulated the levels of pro-inflammatory mediators (COX-2, IL-1β, and TNF-α) and downregulated the production of IL-10, as compared to control cells (Ctrl). However, treatment of RAW 264.7 macrophages with *C. ilicifolia* extract significantly (*p* < 0.05) decreased the levels of pro-inflammatory mediators (IL-1β COX-2, and TNF-α) in a dose-dependent manner (Figure 3A,C,D). In contrast, *C. ilicifolia* extract did not change the level of IL-10 as compared to LPS-treated cells (Figure 3B). Noteworthy, quercetin (25 µM), used as the positive control, was able to downregulate the levels of COX-2, IL-1β, and TNF-α and upregulate the level of IL-10. These data provide evidence that *C. ilicifolia* extract exerts its anti-inflammatory action by suppressing the levels of COX-2, IL-1β, and TNF-α, therefore suggesting that *C. ilicifolia* hydroethanolic extract is a potential source of compounds with anti-inflammatory effects.

### 3.4. Preventive Action of C. ilicifolia Extract on the Production of Reactive Oxygen Species

**Figure 4:**
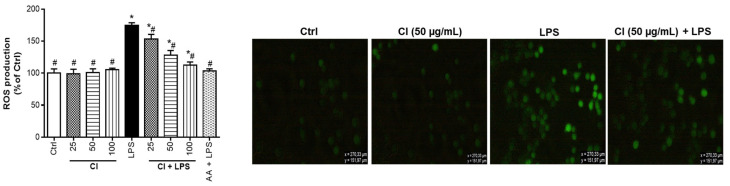
Preventive action of *C. ilicifolia*, and ascorbic acid on the release of reactive oxygen species in LPS-activated RAW 264.7 macrophages. These cells were exposed to non-toxic concentrations of *C. ilicifolia* (CI) and ascorbic acid (AA) at 25 µg/mL for 2 h, and then treated or not with LPS (200 ng/mL) for 24 h. Intracellular ROS levels were measured using the probe DCFH-DA (10 µM), and the cell fluorescence was measured at 485 nm (excitation) and 535 nm (emission). Intracellular ROS levels were expressed as percentage of 0.5% DMSO-treated cells (Ctrl). Each bar represents the mean ± SEM of three replicates (*n* = 3). One-way ANOVA combined Dunnett or Student–Newman–Keuls’s tests was used for data analysis. * *p* < 0.05 vs. Ctrl. ^#^ *p* < 0.05 vs. LPS.

Reactive oxygen species (ROS) are metabolic products emerging from various cells, and they play a key role in the induction and/or development of several diseases through the stimulation of signaling pathways related to oxidative stress [39,40]. The modulation of oxidative stress created by the uncontrolled release of ROS can be monitored either by the antioxidant protection networks within the cells or by the addition of natural antioxidants from medicinal plants or foodstuffs [41]. Therefore, we analyzed the preventive action of *C. ilicifolia* extract on LPS-induced oxidative stress on RAW 264.7 murine macrophages by determining the levels of ROS using DCFH-DA. Figure 4 illustrates the protective effect of *C. ilicifolia* extracts against ROS production. Treatment of RAW 264.7 murine macrophages with *C. ilicifolia* (CI) extract did not induce a significant release of ROS as compared to untreated cells (Ctrl), while the treatment of RAW 264.7 macrophages with LPS induced a significant inflation of ROS production compared to untreated cells (Ctrl). Most importantly, the pre-treatment of RAW 264.7 murine macrophages with *C. ilicifolia* extract (CI + LPS) significantly blocked LPS-induced ROS production. The positive control (ascorbic acid, 25 µg/mL) was also found to prevent LPS-induced ROS production. Ascorbic acid is a known anti-inflammatory and antioxidant compound that has a radical scavenging capacity, including ROS production inhibition at an early stage of their formation through the activation of intracellular antioxidant systems [41]. After determining the percentage inhibition of ROS production by extracts, it was observed that *C. ilicifolia* extract decreased the generation of ROS in a dose-dependent manner with IC_50_ values of 43.07 ± 2.08 µg/mL, respectively (Table 1). These results showed that the antioxidant capacity of *C. ilicifolia* extract was able to reduce the generation of intracellular ROS activated by LPS and further confirmed its antioxidant and anti-inflammatory potency by protecting RAW 264.7 murine macrophages from the deleterious effects of ROS. These data agree with the above-mentioned results from antioxidant assays and suggest potential correlations between the biological activities of *C. ilicifolia* extract and its phytochemical composition. 

### 3.5. Phytochemical Composition of C. ilicifolia

#### 3.5.1. Total Phenolic and Total Flavonoid Contents

It is well known that phenolic compounds and flavonoids improve human health by preventing and treating many diseases. These phytochemicals can be used to reinforce the immune system or shield the skin from ultra-violet (UV) radiation, as well as being interesting sources of bioactive compounds with antibacterial, cardioprotective, anticancer, anti-inflammatory, and antioxidant effects [42,43]. Due to the importance of these phytochemicals and considering the traditional uses of *C. ilicifolia* for the management of several infectious and chronic diseases, the total phenolic (TPC) and flavonoid (TFC) contents of their crude extracts were determined (Table 2). *C. ilicifolia* hydroethanolic leaf extracts were found to have the highest total phenolics (109.32 ± 5.26 mgGAE/g of extract) and flavonoids (23.63 ± 2.03 mg QE/g of extract, respectively). In a qualitative phytochemical screening of these plant extracts, lower quantities of phenolics and flavonoids were reported in *C. ilicifolia* methanolic leaf extracts compared to amounts found in their hydroethanolic extracts [44]. This could be attributed to the polarity of solvents used since it has been confirmed that alcoholic aqueous mixtures are very effective for the extraction of the highest amounts of phenolics and flavonoids [32,45]. Hydro-ethanol (20:80) solvent is highly recommended in the extraction of medicinal plants as it can dissolve both polar and non-polar molecules. Further, our results suggest that the antioxidant activity of *C. ilicifolia* extract is mainly related to the presence of phenolics, while flavonoids are responsible for its anti-inflammatory activity. The antioxidant activity of phenolic compounds is attributed to the availability of free hydroxyl groups and functional groups within aromatic rings having the capacity to donate electrons or hydrogen to oxidant species, thereby forming stable end products or attenuating ROS production [46]. On the other hand, flavonoids are recognized to exert their anti-inflammatory activity by suppressing the release of several mediators, including NO and cytokines, and downregulating the enzyme activity of cyclooxygenases and lipoxygenases, thereby decreasing the level of pro-inflammatory mediators within the cells [47,48].

#### 3.5.2. LC–MS Profile of *C. ilicifolia* Extract

**Figure 5:**
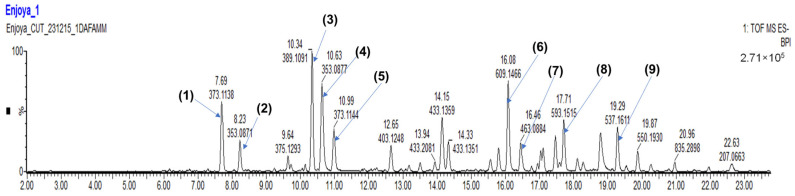
Phytochemical composition of *C. ilicifolia* hydroethanolic leaf extract using liquid chromatography–mass spectrometric (LC–MS). High-resolution UPLC–MS analysis was used for the detection of phytochemicals in *C. ilicifolia* hydroethanolic leaf extract by injecting two microliters into a Waters Cyclic Quadrupole time-of-flight (qTOF) mass spectrometer (MS) connected to a Waters Acquity ultra-performance liquid chromatograph (UPLC) (Waters, Milford, MA, USA). Major compounds detected were (**1**) secologanic acid (RT: 7.69 min), (**2**) chlorogenic acid (3CQA) (RT: 8.23 min), (**3**) monotropein (RT: 10.34 min), (**4**) chlorogenic acid (5CQA) (RT:10.63 min), (**5**) geniposidic acid (RT: 10.99 min), (**6**) rutin (RT: 16.08 min), (**7**) quercetin 3-galactoside (RT: 16.46 min), (**8**) astragalin-7-rhamnoside (RT: 17.71 min), and (**9**) minecoside (RT: 19.29 min).

**Figure 6:**
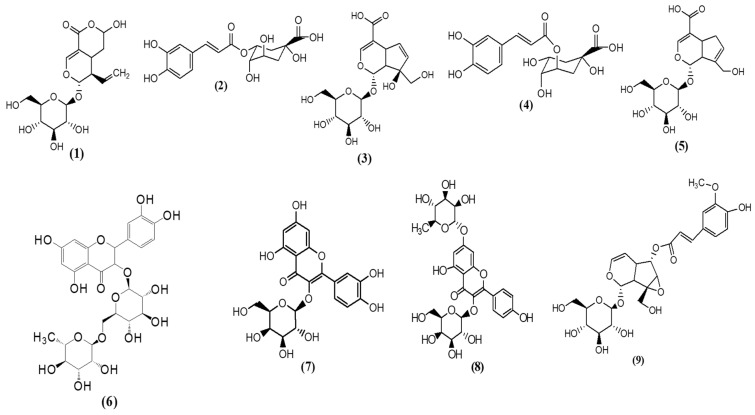
Chemical structures of identified compounds in *C. ilicifolia* hydroethanolic leaf extract. Secologanic acid (**1**), chlorogenic acid (3CQA) (**2**), monotropein (**3**), chlorogenic acid (5CQA) (**4**), geniposidic acid (**5**), rutin (**6**), quercetin 3-galactoside (**7**), astragalin-7-rhamnoside (**8**), and minecoside (**9**).

Based on its consistent potency in all antioxidant and anti-inflammatory activities, *C. ilicifolia* extract was analyzed using the LC–MS method. Thirty compounds were tentatively identified by matching their MS1 and MS2 spectra with search databases. The most abundant compounds were (**1**) secologanic acid (RT: 7.69 min), (**2**) chlorogenic acid (3CQA) (RT: 8.23 min), (**3**) monotropein (RT: 10.34 min), (**4**) chlorogenic acid (5CQA) (RT:10.63 min), (**5**) geniposidic acid (RT: 10.99 min), (**6**) rutin (RT: 16.08 min), (**7**) quercetin 3-galactoside (RT: 16.46 min), (**8**) astragalin-7-rhamnoside (RT: 17.71 min), and (**9**) minecoside (RT: 19.29 min) (Table 3; Figure 5) with their chemical structures being presented in Figure 6. The detection of these compounds implied that they may play a key role in the anti-inflammatory and antioxidant activities of *C. ilicifolia*.

Rutin (8342 mg/L), a flavonoid that is found abundantly in many plants, had the highest amount in *C. ilifolia* extract. Preclinical studies have proven that rutin exerts strong antioxidant properties by inhibiting xanthin oxidase, which is an enzyme implicated in ROS production [49,50,51]. Rutin also increases the production of glutathione (GSH) and strengthens cellular oxidative protection systems through the enhancement of antioxidant enzymes such as superoxide dismutase (SOD) and catalase (CAT) [52]. Additionally, rutin ameliorates inflammation by inhibiting the release of IL-1β, IL-6, NO, TNF-α, COX-2, interferon-gamma (IFN-γ), monocyte chemoattractant protein-1 (MCP-1), and downregulating the activation of NF-κB and mitogen activated protein kinase (MAPK) signaling pathways in palmitate or LPS-activated RAW 264.7 murine macrophages and animal-induced inflammatory models [53,54,55,56]. Other flavonoids found in *C. ilicifolia* were astragalin-7-rhamnoside (4050 mg/L) and quercetin 3-galactoside (1539 mg/L). The anti-inflammatory and antioxidant potentials of astragalin-7-rhamnoside are not yet fully studied. However, quercetin 3-galactoside (also known as hyperoside) is known to suppress the levels of NO, TNF-α, and IL-6 by downregulating NF-κB translocation and inhibitory κB-α (IκB-α) degradation [57]. Hyperoside also exerts its antioxidant potency by hunting radicals such as ABTS^•+^ and DPPH^•^. It reduces oxidative stress by alleviating lipid peroxidation and intracellular ROS production via the modulation of nuclear factor erythroid 2-related factor 2 (Nrf2)/antioxidant response element (ARE) antioxidant pathways [58,59]. The second most abundant compound in *C. ilifolia* extract was secologanic acid (5342 mg/L), an iridoid glycoside, which is reported to exhibit its anti-inflammatory action by suppressing the expression of protein kinase B (AkT) and p-AkT that mediate the activation of downstream NF-κB to stimulate the secretion of pro-inflammatory mediators such as TNF-α, therefore aggravating inflammation [60]. Other iridoid glycosides detected in *C. ilicifolia* were monotropein (3973 mg/L) and geniposidic acid (1497 mg/L). Monotropein is known to prevent cell apoptosis by decreasing ROS production and inhibiting LPS-stimulated release of inflammatory mediators such as COX-2, iNOS, IL-1β, and TNF-α by downregulating the phosphorylation and degradation of IκB-α. Consequently, this action resulted in the translocation of NF-κB, which plays an important role in the initiation and progression of oxidative stress and inflammatory responses [61,62]. Monotropein is also able to alleviate H_2_O_2_-induced inflammation and oxidative stress by depleting the level of malondialdehyde and enhancing the activity of SOD and glutathione peroxidase (GSH-Px) [63]. Geniposidic acid (GPA) is reported to relieve oxidative stress by triggering the Akt/Nrf2 pathway [64], and it also reduces ROS production by inhibiting xanthine oxidase [65]. Additionally, GPA exerts its anti-inflammatory activity by suppressing the levels of IL-6, IL-1β, and TNF-α through the suppression of MAPK and NF-κB signaling pathways [66,67]. Another compound that was found in considerable amounts in *C. ilicifolia* extract was minecoside (3441 mg/L), which is reported to exhibit an antioxidative effect in an oxygen radical absorbance capacity assay [68]. Two quinic acid derivatives, i.e., chlorogenic acids (3CQA and 5CQA, 1191 mg/L and 2375 mg/L, respectively), were also found in the *C. ilicifolia* extract. Chlorogenic acids have been shown to lessen inflammation and oxidative stress by diminishing ROS, hydroxyl, and superoxide radicals [69,70], and they can also attenuate the generation of pro-inflammatory mediators [71,72]. The presence of all the above-mentioned compounds, which may act synergistically or additively to contribute to the anti-inflammatory and antioxidant potentials of *C. ilicifolia*, may justify its strong efficacy in scavenging radicals such as DPPH^•^, ABTS^•+^ and ROS, as well as suppressing the levels of COX-2, 15-LOX, IL-1β, and TNF-α. These results therefore support the medicinal uses of *C. ilicifolia* against oxidative stress and inflammatory-related diseases. However, the pharmacological activities of some compounds identified in *C. ilicifolia* are not yet studied, and several unrevealed compounds are still not elucidated, therefore launching further research directives regarding the bioassay-guided isolation of anti-inflammatory and antioxidant compounds from this plant species.

## 4. Conclusions

Overall, data obtained from the current study demonstrated that *C. ilicifolia* hydroethanolic extract exerts its anti-inflammatory and antioxidant effects by lowering the levels of radicals such as ABTS^•+^ and DPPH^•^ and suppressing ROS production and the release of COX-2, IL-1β, NO, and TNF-α in LPS-activated RAW 264.7 murine macrophages. Moreover, *C. ilicifolia* extract efficiently inhibited the enzymatic activity of 15-LOX, a key enzyme in the production of leukotrienes, which mediate the inflammatory process. Moreover, based on the fact that the release of NO and cytokines in LPS-stimulated cells is activated by ROS production, further investigation on other molecular markers such as iNOS, GSH, CAT, and SOD is needed to fully elucidate the mechanism of action of *C. ilifolia* extract and to support its potential application for the management of oxidative stress and inflammatory conditions. However, due to its richness in several identified compounds with recognized anti-inflammatory and antioxidant activities, the *C. ilicifolia* extract depicted strong efficacy in all assays, thereby indicating this plant species to be a good potential alternative to conventional drugs against oxidative stress and inflammatory-related diseases. These results open doors for further investigations regarding the pharmacological and phytochemical analyses of *C. ilicifolia* for the discovery of novel therapeutics and the development of plant-based products against inflammation and oxidative stress-related conditions.

## Figures and Tables

**Table 1:** Antioxidant and anti-inflammatory potentials of *C. ilicifolia* hydroethanolic plant extract.

Plant Name	IC_50_ (µg/mL)					
	DPPH	ABTS	FRAP	ROS	NO	15-LOX
*C. ilicifolia*	31.61 ± 1.35	21.29 ± 1.36	>200	43.07 ± 2.08	21.10 ± 1.20	40.28 ± 1.35
Ascorbic acid	4.99 ± 0.20 *	4.64 ± 0.85 *	26.68 ± 1.34 *	5.76 ± 0.87 *	n.d.	n.d.
Quercetin	n.d.	n.d.	n.d.	n.d.	2.11 ± 1.01 *	n.d.
Gallic acid	n.d.	n.d.	n.d.	n.d.	n.d.	22.08 ± 1.96 *

Data are presented as the mean of three replicates ± standard error of the mean (SEM); * mean statistically significantly different (*p* < 0.05). IC_50_: concentration required to inhibit 50% of each biological activity compared to negative controls. n.d.: not determined.

**Table 2:** Extraction yield and phytochemical contents of hydroethanolic extracts of selected plants.

Plant Name	% Extraction Yield (g Extract/100 g Dry Material)	Phenolic Content (mgGAE/g Extract)	Flavonoid Content (mgQE/g Extract)
*C. ilicifolia*	15.66	109.32 ± 5.26	23.63 ± 2.03

Data are presented as the mean of three replicates ± standard error of mean (SEM); QE: quercetin equivalent, GAE: gallic acid equivalent.

**Table 3:** Natural compounds detected in *C. ilicifolia* hydroethanolic leaf extract, using negative mode ionization liquid chromatography–mass spectrometric analysis.

Peak N°	RT (min)	[M-H]-(*m*/*z*)	Tentative Assignment (Compound Name)	Ontology	Molecular Formula	Total Score	Peak Height Intensity	Conc. in Extract vs. 3CQA (mg/L)
1	6.77	315.07	Gentesic acid 5-O-glucoside	Phenolic glycosides	C_13_H_16_O_9_	7.20	2096	77
2	7.28	473.13	Trans-Caffeic acid [apiosyl-(-glucosyl] ester	Hydroxycinnamic acid glycosides	C_20_H_26_O_13_	6.32	4933	182
3	7.69	373.11	**Secologanic acid**	Terpene glycosides	C_16_H_22_O_10_	7.13	144,505	**5342**
4	8.23	353.08	**Chlorogenic acid 3CQA**	Quinic acids and derivatives	C_16_H_18_O_9_	7.83	13,644	**1191**
5	9.23	373.11	Geniposidic acid	Iridoid O-glycosides	C_16_H_22_O_10_	7.55	6376	236
6	9.64	375.12	Loganic acid	Iridoid O-glycosides	C_16_H_24_O_10_	8.61	32,220	504
7	10.34	389.10	**Monotropein**	Iridoid O-glycosides	C_16_H_22_O_11_	7.46	107,470	**3973**
8	10.63	353.08	**Chlorogenic acid 5CQA**	Quinic acids and derivatives	C_16_H_18_O_9_	8.82	64,238	**2375**
9	10.99	373.11	**Geniposidic acid**	Iridoid O-glycosides	C_16_H_22_O_10_	7.63	40,505	**1497**
10	13.51	403.12	Oleoside 11-methyl ester	Terpene glycosides	C_17_H_24_O_11_	7.43	17,541	648
11	13.79	435.22	Amphipaniculoside E	Fatty acyl glycosides of mono- and disaccharides	C_20_H_36_O_10_	5.52	4102	152
12	14.51	625.14	Quercetin 3-glucosyl-galactoside	Flavonoid-3-O-glycosides	C_27_H_29_O_17_	7.22	625	23
13	15.47	581.22	(7′R)-(+)-Lyoniresinol 9′-glucoside	Lignan glycosides	C_28_H_38_O_13_	6.25	636	24
14	15.81	609.14	Quercetin robinobioside	Flavonoid-3-O-glycosides	C_27_H_30_O_16_	7.07	48,294	785
15	16.08	609.14	**Rutin**	Flavonoid-3-O-glycosides	C_27_H_30_O_16_	8.09	225,648	**8342**
16	16.14	463.08	Kaempferol 3-alpha-L-arabinopyranoside	Flavonoid-3-O-glycosides	C_20_H_18_O_10_	6.13	4716	174
17	16.46	463.08	**Quercetin 3-galactoside**	Flavonoid-3-O-glycosides	C_21_H_20_O_12_	7.90	41,638	**1539**
18	17.15	515.12	3,4-Dicaffeoylquinic acid	Isoflavonoid O-glycosides	C_25_H_24_O_12_	6.67	800	30
19	17.46	503.25	4,7-Megastigmadiene-3,9-diol-9-[apiosyl-(1-6)-glucoside]	Fatty acyl glycosides of mono- and disaccharides	C_24_H_40_O_11_	5.94	73,694	724
20	17.59	515.12	3,5-Dicaffeoylquinic acid	Quinic acids and derivatives	C_25_H_24_O_12_	6.11	18,935	700
21	17.71	593.15	**Astragalin 7-rhamnoside**	Flavonoid-7-O-glycosides	C_27_H_30_O_15_	8.12	109,553	**4050**
22	18.11	447.09	Astragalin	Flavonoid-3-O-glycosides	C_21_H_20_O_11_	8.09	22,404	828
23	18.27	447.09	Quercitrin	Flavonoid-3-O-glycosides	C_21_H_20_O_11_	6.76	12,741	471
24	18.79	515.11	4,5-Dicaffeoylquinic acid	Quinic acids and derivatives	C_25_H_24_O_12_	6.48	25,615	947
25	19.29	537.16	**Minecoside**	Coumaric acids and derivatives	C_25_H_30_O_13_	5.95	93,070	**3441**
26	20.25	431.09	Kaempferol rhamnoside	Flavonoid O-glycosides	C_21_H_20_O_10_	8.03	9675	358
27	20.78	535.18	8-Hydroxypinoresinol 8-glucoside	Lignan glycosides	C_26_H_32_O_12_	7.27	4710	174
28	20.96	835.28	Sylvestroside II	Iridoid O-glycosides	C_35_H_50_O_20_	5.42	8788	325
29	21.95	759.23	Adinoside D;(-)-Adinoside D	Terpene glycosides	C_33_H_44_O_20_	6.58	9653	357
30	22.63	207.06	6-Methoxymellein	2-benzopyrans	C_11_H_12_O_4_	7.60	3160	117

RT: retention time; 3CQA: chlorogenic acid. Compounds indicated with bold are found in higher concentration in the plant species.

## Data Availability

The original contributions presented in the study are included in the article, and further details on these data will be available from the corresponding authors on request.

## Supplementary Materials

The following supporting information can be downloaded at: https://www.mdpi.com/article/10.3390/cimb46090573/s1, Figure S1: Non-linear regression graphs for the determination of IC_50_ values of *C. ilicifolia* hydroethanolic leaf extract in *ABTS* (A) and DPPH (B) assays. Figure S2: Non-linear regression graphs for the determination of IC_50_ values of *C. ilicifolia* hydroethanolic leaf extract (A) and gallic acid (B) in 15-LOX inhibitory assay. Figure S3: Non-linear regression graphs for the determination of IC_50_ values of *C. ilicifolia* (A), and quercetin (B) in the NO production inhibitory assay.
