# Supplementary material for: Investigating the Phytochemical Composition, Antioxidant, and Anti-Inflammatory Potentials of Cassinopsis ilicifolia (Hochst.) Kuntze Extract against Some Oxidative Stress and Inflammation Molecular Markers
Source: Curr Issues Mol Biol. 2024 Sep 1;46(9):9639–58. doi: 10.3390/cimb46090573 (PMC11429818; doi:10.3390/cimb46090573)
Supplement: Supplementary file 1 [file cimb-46-00573-s001.zip › cimb-3178873-supplementary.pdf]

Supplementary information

Investigating the Phytochemical Composition, Antioxidant, and Anti-Inflammatory Potentials of *Cassinopsis ilicifolia* (Hochst.) Kuntze Extract against Some Oxidative Stress and Inflammation Molecular Markers

Emmanuel Mfotie Njoya [1,*], Lyndy J. McGaw [2] and Tshepiso J. Makhafola [1,*]

[1] Centre for Quality of Health and Living, Faculty of Health and Environmental Sciences, Central University of Technology, Bloemfontein 9300, Free State, South Africa
[2] Phytomedicine Programme, Department of Paraclinical Sciences, Faculty of Veterinary Science, University of Pretoria, Private Bag X04, Onderstepoort, Pretoria 0110, Gauteng, South Africa; lyndy.mcgaw@up.ac.za
* Correspondence: mfotierfr@yahoo.fr or enjoya@cut.ac.za (E.M.N.); jmakhafola@cut.ac.za (T.J.M.)

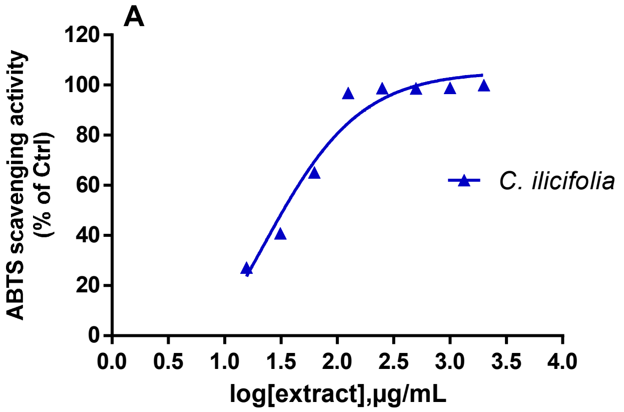

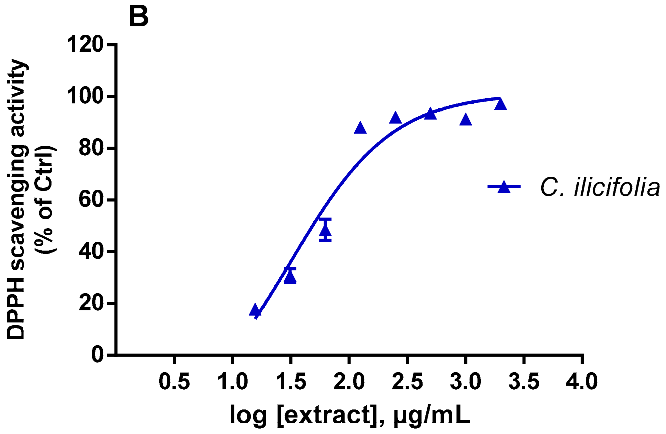

Figure S1: Non-linear regression curves for IC_{50} determination of *C. ilicifolia* hydroethanolic leaf extract in ABTS (**A**) and DPPH (**B**) assays.

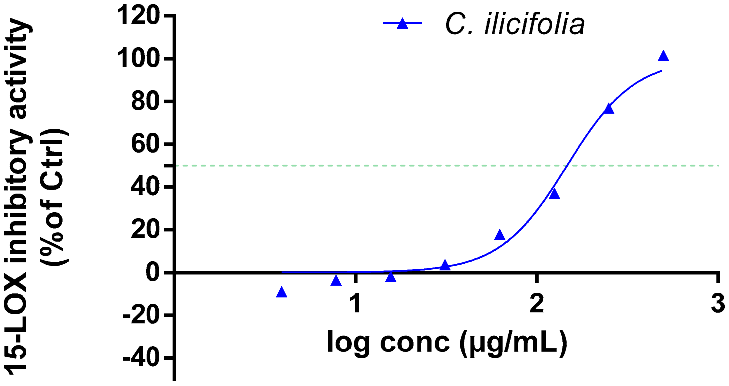

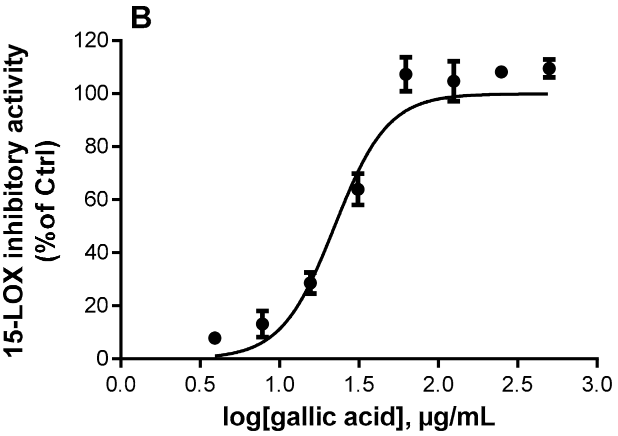

Figure S2: Non-linear regression curves for IC_{50} determination of *C. ilicifolia* hydroethanolic leaf extracts (**A**) and gallic acid (**B**) in 15-lipoxygenase (15-LOX) inhibitory assay.

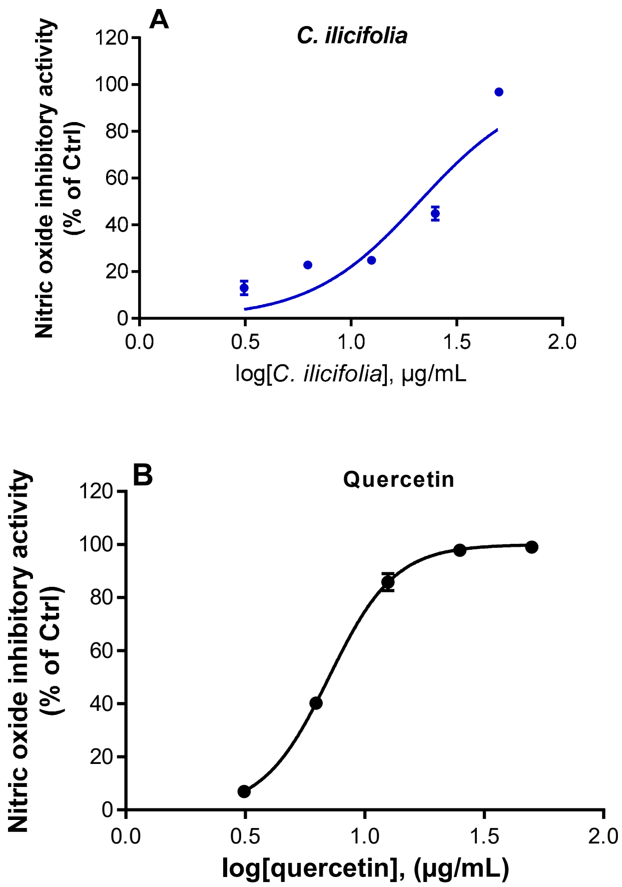

Figure S3: Non-linear regression curves for IC_{50} determination of *C. ilicifolia* (**A**), and quercetin (**B**) in nitric oxide (NO) production inhibitory assay.
